# Is Promiscuous CALB a Good Scaffold for Designing New Epoxidases?

**DOI:** 10.3390/molecules201017789

**Published:** 2015-09-25

**Authors:** Isabel Bordes, José Recatalá, Katarzyna Świderek, Vicent Moliner

**Affiliations:** 1 Departament de Química Física i Analítica, Universitat Jaume I, Castellón 12071, Spain; E-Mails: bordes@uji.es (I.B.); al189587@alumail.uji.es (J.R.); 2 Institute of Applied Radiation Chemistry, Lodz University of Technology, Lodz 90-924, Poland

**Keywords:** *Candida antarctica* lipase B, CALB, epoxide hydrolase, sEH, reaction mechanism, trans-diphenylpropene oxide, enzyme promiscuity, catalysis, quantum cluster models

## Abstract

*Candida Antarctica* lipase B (CALB) is a well-known enzyme, especially because of its promiscuous activity. Due to its properties, CALB was widely used as a benchmark for designing new catalysts for important organic reactions. The active site of CALB is very similar to that of soluble epoxide hydrolase (sEH) formed by a nucleophile-histidine-acid catalytic triad and an oxyanion hole typical for molecular structures derived from processes of α/β hydrolases. In this work we are exploring these similarities and proposing a Ser105Asp variant of CALB as a new catalyst for epoxide hydrolysis. In particular, the hydrolysis of the trans-diphenylpropene oxide (t-DPPO) is studied by means of quantum cluster models mimicking the active site of both enzymes. Our results, based on semi-empirical and DFT calculations, suggest that mutant Ser105Asp CALB is a good protein scaffold to be used for the bio-synthesis of chiral compounds.

## 1. Introduction

Epoxides are important molecules for producing chiral compounds. Because of their chemical versatility and ability to react readily with halides, carbon, nitrogen, oxygen, or sulfur nucleophiles, epoxides became crucial intermediate products in organic synthesis [1]. Epoxides are three-membered cyclic ethers that have specific reactivity patterns characterized by their highly polarized oxygen–carbon bonds in addition to a highly strained ring [2]. In recent years enormous efforts have been done into developing methodologies for preparing enantio-pure forms of epoxides. This purpose has been achieved by applying new chemical [3] and biocatalytic [4] procedures. Biocatalytic conversion of epoxides can proceed by, for instance, conjugation of thiol cofactors, nucleophilic ring opening, or hydrolysis. The last process is, in fact, the subject of the present work.

Herein we focus on the epoxide hydrolases (EHs, E.C.3.3.2.3) that serve as a catalyst to transform epoxide into the corresponding 1,2-diol by addition of a water molecule. In fact, mammalian EHs were widely studied mostly because of their biological functions. The main roles of EHs in mammalian organisms are detoxification, catabolism, and regulation of signaling molecules. However, EHs have mainly garnered interest because of their potential applications in chiral chemistry [1,5].

Soluble epoxide hydrolase (sEH) hydrolyses a broad range of substrates such as gem-di-, trans-di-, cis-di, tri-, and tetra-substituted epoxides [6]. However, for the typical *in vitro* purposes, the substrate is trans-diphenylpropene oxide (t-DPPO), and thus it will be used as a substrate in the present study. Recently, it was found that sEH plays an important role in the regulation of blood pressure and inflammation [7,8,9,10,11,12,13], which also makes it a good target for designing drugs to be used in the treatment of several diseases, including several aspects of cardiovascular diseases such as inflammation, hypertension, cardiac hypertrophy, and atherosclerosis, or kidney failure [9,13,14,15,16].

**Scheme 1:**
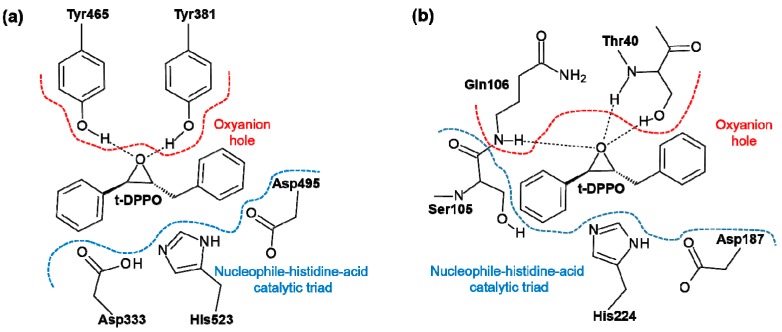
Active site of (**a**) sEH and (**b**) CALB enzymes together with bound (*R*,*R*)-*trans*-diphenylpropene oxide (t-DPPO).

sEH belongs to the α/β hydrolase fold family of proteins, which are characterized by a Nucleophile-His-Acid catalytic triad evolved to efficiently operate on substrates with different chemical composition or physicochemical properties and in various biological contexts [17]. In the case of human sEH, this triad is formed by Asp333, His523, and Asp495, as presented in Scheme 1a. Moreover, an “oxyanion hole” is formed in the active sites by two tyrosine residues, Tyr-381 and Tyr-465, which are assumed to stabilize the tetrahedral intermediate by protonation or hydrogen bonding interaction of the oxygen atom of the epoxide [18]. The importance of these two residues for the catalytic process was proven experimentally when a 90% decrease in sEH activity was observed when one of the tyrosine residues was mutated to phenylalanine [19].

The structure of the sEH active site is very similar to other enzyme from the α/β hydrolases family named *Candida antarctica* lipase B (CALB), widely studied because of its high catalytic promiscuity [20]. The mechanism of primary reaction of CALB, which is the hydrolysis of ester bonds, was recently studied experimentally [21] and theoretically described [22], providing interesting insights into the functions of residues in the active site. It has been suggested that CALB can be used as an efficient catalyst of biotransformation typical for a carboxylic acid like esterase, thioesterase, peptidase, dehalogenase, epoxide hydrolase, or halo peroxidase, or having the ability to cleave and form C–C bonds [17,23,24]. Moreover, CALB has been also used as a catalyst in ring-opening polymerization reactions [25]. Interestingly, it was shown that both wild-type and Ser105Ala-mutated variants of CALB are able to catalyze direct epoxidation of an α,β-unsaturated aldehyde with hydrogen peroxide [26]. In the present work, we want to find out if CALB could be used as a scaffold for new catalysts to serve as an epoxide hydrolase.

The active site of CALB contains a Nucleophile-His-Acid catalytic triad formed by Ser105, His224, and Asp187. As in sEH, there is an oxyanion hole in the active site of CALB formed by Thr40 and Gln106 residues. The similarities between the sEH and CALB active sites are indicated on Scheme 1.

It is generally assumed that the mechanism of hydrolysis of epoxides catalyzed by sEH to the corresponding diols is known. It was demonstrated using experimental techniques that the epoxides can be opened by direct attack of a nucleophile on the epoxide ring or via an intermediate in which a covalent link between the enzyme and the substrate is formed. This variant of the mechanism, in which the nucleophile Asp333 was involved in the reaction, was recently confirmed by theoretical studies performed by Mulholland and co-workers [27]. However, an important question about the origin of the hydrogen that binds to the oxygen of the epoxide after formation of the oxyanion intermediate remains unanswered. Some possible mechanisms have already been proposed, including proton transfer from one of the tyrosine residues that forms the oxyanion hole [28], or direct transfer of a proton from His523 [29]. Nevertheless, these proposals were never explored by theoretical studies. Thus, one of the targets of this work is to dispel these doubts. Recently, a proposal of proton diffusion to the solvent was suggested from a computational, structural, and kinetic study on potato epoxide hydrolase [30].

**Scheme 2:**
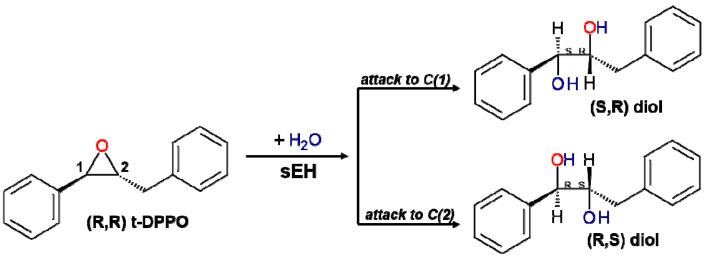
Possible products of hydrolysis of (*R*,*R*)-trans-diphenylpropene (t-DPPO).

In the case of hydrolysis of t-DPPO, attacks on both the C1 or C2 carbon atom of epoxide are possible, resulting in two different enantiomers, as presented in Scheme 2.

**Figure 1:**
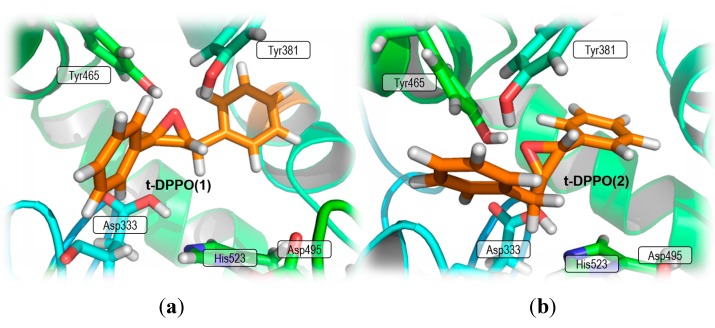
Active site of sEH with bound substrate (**a**) t-DPPO(1) and (**b**) t-DPPO(2) orientation.

However, sEH shows regioselectivity by preferentially attacking the reactive C1 carbon atom rather than C2 with a 97:3 selectivity ratio [31]. As was observed in previous theoretical studies [27], this tendency is observed in both possible orientations of substrate t-DPPO(1) and t-DPPO(2), which are related to the position of the substrate relative to the arrangement of residues in the active site of the enzyme, as presented in Figure 1.

In the present work we are proposing a new catalyst for epoxide hydrolysis reaction based on the structure of the active site of CALB. In order to examine our proposal, the mechanism of the hydrolysis of (*R*,*R*)-t-DPPO in active sites of sEH and a variant of CALB is described using quantum cluster theoretical models.

## 2. Results and Discussion

### 2.1. t-DPPO Hydrolysis Catalyzed by sEH

**Scheme 3:**
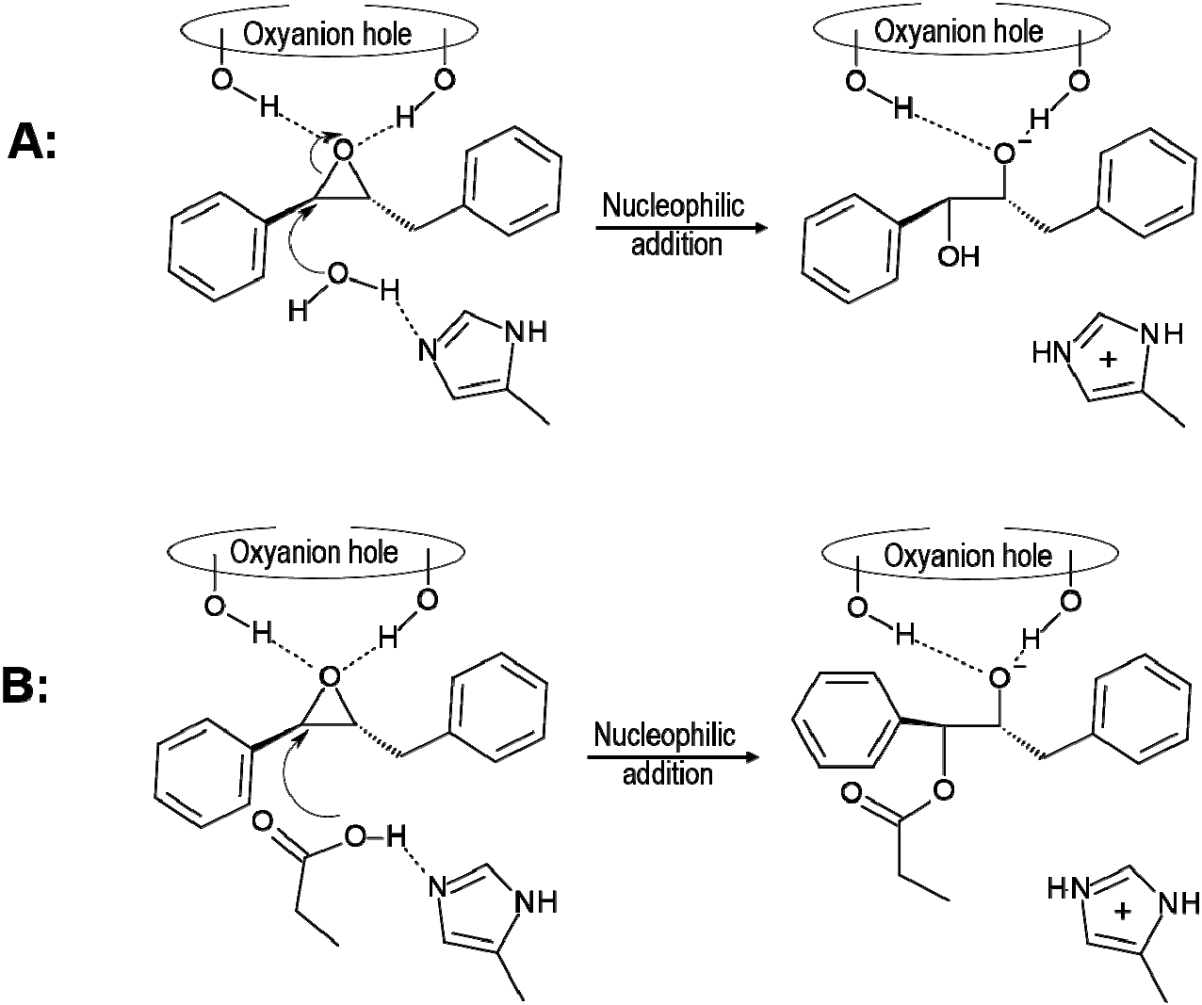
Possible mechanism of nucleophilic attack to the carbon C1 of t-DPPO(1) epoxide by (**A**) a water molecule and (**B**) Asp333.

The preparations of the theoretical models require knowledge about the protonation state of titratable residues. Nowadays, fast empirical prediction of pKa algorithm (PROPKA software) [32,33,34,35] makes the analysis of protonation states for these residues computationally available. The protonation state of some residues, especially those forming the catalytic triad, seems to be still an open question of debate [29,30,36,37]. In the case of sEH, it was previously observed that protonation of His523 meaningfully influences the rate constant of nucleophilic attack, and it was assumed that this residue has to be protonated in order to reduce the barrier of this step [27]. However, our analysis of pKa results shows that, after substrate binding, the His523 is surely not protonated at pH for sEH activity, which is 7.0–7.5 [6]. This result is in agreement with recent studies on potato sEH [30] and previous studies of Himo and co-workers on human sEH [36,37]. Moreover, it is observed that Asp333, which plays the role of the nucleophile in the studied reaction, has a high pKa value (over 10) (see Table S1 in the Supplementary Materials), which indicates that at the beginning of the hydrolysis it must be protonated, a proposal not suggested in previous studies. Our results indicate that Asp333 has to be activated by transferring the proton and thus acting as the nucleophile that attacks the carbon of epoxide. This step is then part of the full reaction path. Nevertheless, the lack of proton on His523 opens another possible mechanism of this reaction in which, as previously suggested [38], instead of Asp333 a water molecule can be activated by His523 and subsequently it can attack one of the carbon atoms of the epoxide ring. Thus, both scenarios, (A) attack of water and (B) attack of Asp333, will be taken into consideration in the present study, as depicted in Scheme 3.

In the case of mechanism A, the nucleophilic attack can take place concurrently with the proton transfer from a water molecule to Nε-His523. This possibility was investigated by exploring the corresponding potential energy surface (PES), where the antisymmetric combination of distances defined between the oxygen of the water molecule and the carbon atom of epoxide, and between this carbon atom and the oxygen atom of epoxide (d(O^wat^–C1)-d(O^ep°x^–C1)) was used as one of the distinguished reaction coordinates. The other one was defined as the antisymmetric combination of distances defining the position of the transferred hydrogen atom between the oxygen atoms of the water molecule and the nitrogen atom in position ε of His523 (d(O^wat^–H^wat^)-d(Nε–H^wat^)).

**Figure 2:**
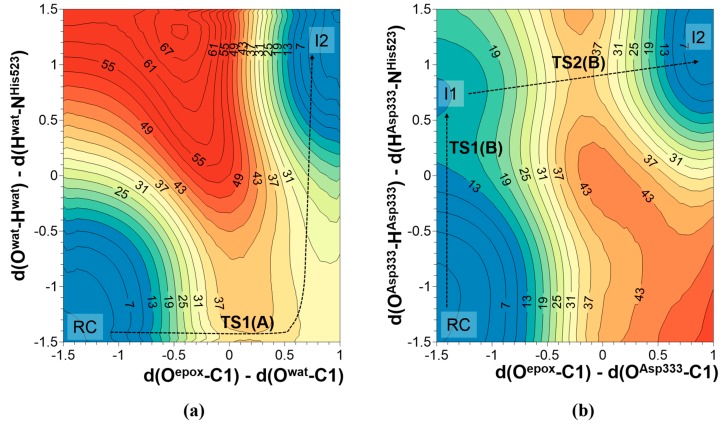
AM1 PES of nucleophilic attack to C1 of t-DPPO(1) by (**a**) a water molecule; and (**b**) Asp333. Distances are in Å and values on iso-energetic lines in kcal·mol^−1^.

In order to explore the second possibility of nucleophilic addition, the nucleophilic attack to C1 of t-DPPO(1) by Asp333, a similar PES was explored. In this case, the antisymmetric combination of distances between oxygen from Asp333 and the carbon of epoxide and between this carbon atom and the oxygen of epoxide (d(O^Asp333^–C1)-d(O^ep°x^–C1)) was used as one of the coordinates, while the antisymmetric combination of distances describing the transfer of hydrogen atom from oxygen of Asp333 to the nitrogen in position ε of His523 (d(O^Asp333^–H^Asp333^)-d(Nε–H^Asp333^)) were controlled as the second reaction coordinate. The resulting PESs are shown in Figure 2. In mechanism A, the nucleophile attack of water occurs simultaneously with proton transfer to Nε-His523. The estimated potential energy barrier for this step is 38.1 kcal·mol^−1^ at AM1 level and 28.5 kcal·mol^−1^ after correction at M06-2X level. On the other side, mechanism B takes place through a step-wise process whose rate-determining step, the attack of the deprotonated oxygen atom of Asp333 to the carbon atom of the epoxide ring, presents an energy barrier of 39.5 kcal·mol^−1^ at AM1 level but is dramatically reduced to 20.3 kcal·mol^−1^ when recomputed at M06-2X/6-31+G(d,p) level. Thus, our simulations predict mechanism B as the kinetically favorable mechanism and thus we will focus on the next steps of this mechanism. This conclusion is in agreement with experimental studies based on solvent KIEs that confirmed the role of Asp333 residue as the attacking nucleophile since the heavy ^18^O oxygen was incorporated into the Asp333 of sEH [36]. Moreover, kinetic measurements done for Asp333Ser mutated sEH resulted in a total loss of activity, again indicating the participation of this residue in the hydrolysis of epoxides [39]. Moreover, the obtained value for the energy barrier is in good agreement with the free energy barriers deduced from experimental kinetic measurements at 300 K using the transition state theory (rate constant between 0.09 and 0.75 s^−1^) [40] for the nucleophilic addition step of hydrolysis of chalconeoxide by sEH. This species can be considered very closely related to the herein-studied t-DPPO substrates. Interestingly, previous theoretical studies for this step resulted in underestimated values of energy barriers equal to 7.8 kcal·mol^−1^ for β-methyl-styrene oxide [32,33,34,35] or 9.7 kcal·mol^−1^ for t-DPPO epoxide [27]. In our opinion, this difference comes from the assumption that the reaction pathway begins with negatively charged Asp333, and thus the first step of proton transfer from Asp333 to Nε-His523 was excluded from proposed mechanisms. Keeping in mind these promising results, the study was repeated for the nucleophilic attack of Asp333 to epoxide carbon C1 with the (2)-DPPO orientation, and on the C2 carbon atom of epoxide in the two different orientations of substrate. The results are summarized in Table 1.

**Table 1:** Relative potential energies obtained at AM1 level related to reactant complex (RC) for nucleophilic attack of Asp333 to epoxide carbons C1 or C2 in two different orientations of substrate in the active site of sEH. Values in parentheses correspond to relative energies of TS2 to I1. Results are given in kcal·mol^−1^.

	(1)-DPPO				(2)-DPPO			
	C1		C2		C1		C2	
	Cluster	QM/MM ^a^	Cluster	QM/MM ^a^	Cluster	QM/MM ^a^	Cluster	QM/MM ^a^
**TS1**	14.0	-	14.0	-	16.3	-	16.3	-
**I1**	11.4(0.0)	0.0	11.4	0.0	12.7	0.0	12.7	0.0
**TS2**	39.9(28.5)	23.5	41.5(30.1)	24.5	42.5(29.8)	18.4	40.2(27.5)	27.6

^a^: theoretical values obtained at AM1/MM level by Mulholland and co-workers [27].

Based on the obtained energy barriers for the nucleophilic attack of Asp333 on either the C1 or C2 of epoxide based on our cluster models of the sEH active site, it is difficult to reach conclusions about its enantioselectivity. Comparing our energy barriers presented in Table 1 with the QM/MM theoretical data previously published by Mulholland and co-workers [27], it seems that in the case of t-DPPO(1), some agreement can be found showing that an attack on the C1 carbon of epoxide is more favorable. However, in case of the second orientation t-DPPO(2) we observe opposite tendency. Nevertheless, the differences of energy barriers are too small and thus it is impossible to obtain clear conclusions. It seems that the selectivity of sEH is influenced not only by the shape of the active site itself but by the relation to the global influence of the rest of the protein, probably including the binding effect. Thus, to explore this feature of sEH, QM/MM studies are required. Similar conclusions can be derived from comparison between both orientations of the substrate.

After the first step of the reaction was studied, in which transformation of epoxide to oxyanion tetrahedral intermediate occurs, we focused on the second part of the mechanism, which is still an unsolved problem. The relevance of this step is evident since it was shown by experimental measurements that this step of the reaction, in which the oxyanion intermediate is transformed into a diol, seems to be the rate-limiting step.

**Scheme 4:**
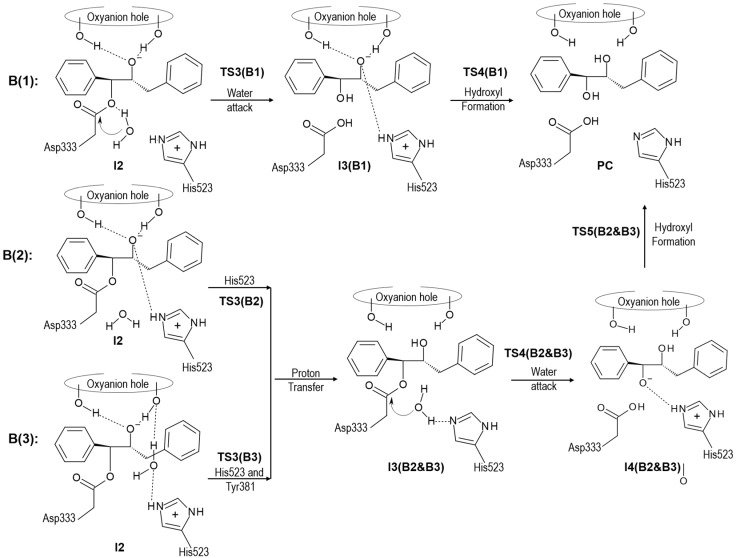
Possible scenarios of hydroxyl formation from oxyanion tetrahedral intermediate of t-DPPO(1) in active site of sEH.

According to the obtained results after the nucleophilic addition, the oxyanion intermediate is formed and the protonation state of His523 is changed (from neutral in reactants to positively charged form in the intermediate). The presence of a hydrogen atom in Nε-position does not allow His523 to activate a water molecule in order to attack the carbon of Asp333 and thus alternative scenarios, depicted in Scheme 4, must be considered. In the first one, mechanism B1, we assumed that water directly attacks the carbon atom of Asp333 with a simultaneous proton transfer to the oxygen of the new ether bridge formed between Asp333 and the C1 carbon atom of the epoxide. After this attack, a final proton transfers from Nε-histidine to the oxygen of epoxide occurs and hydroxyl is formed. In the second possibility, mechanism B2 and B3, the reverse order is explored. In mechanism B2, it is assumed that the proton from Nε-His523 is transferred to the oxygen of epoxide, resulting in neutral histidine, which will then be prepared for the last step of the di-hydroxyl formation process. During the second step, a water molecule is activated by transferring its proton to His523 concomitantly with the attack on the carbon of Asp333. Subsequently, the last proton transfers from Nε-His523 to oxygen bound to the C1 carbon of epoxide, resulting in the expected product. As observed, the difference between mechanism B2 and B3 is the result of different proposals of the origin of hydrogen that is transferred to the negatively charged oxygen atom of epoxide formed in the first step of the process. Thus, in mechanism B3 the proton is transferred from His523 through a water molecule and Tyr381.

**Figure 3:**
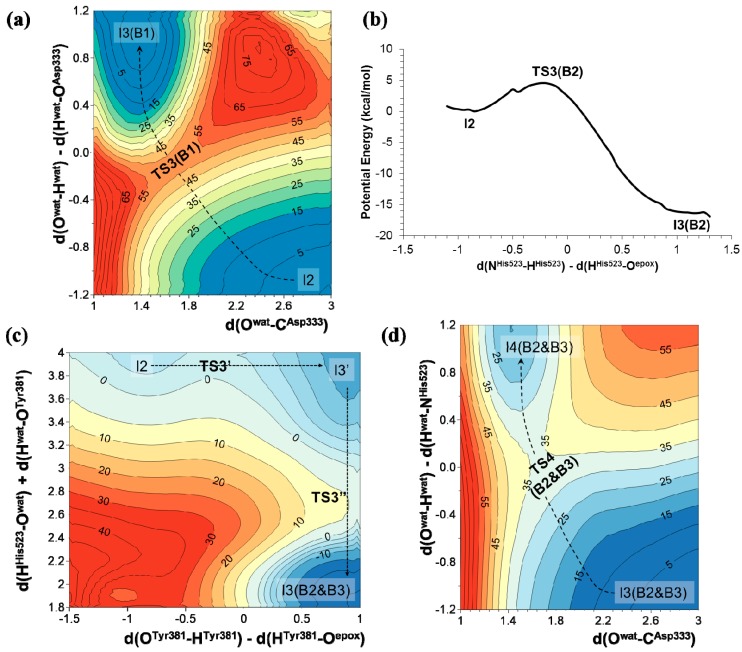
AM1 PESs for hydroxyl formation from oxyanion tetrahedral intermediate of t-DPPO(1) through different possible mechanisms: (**a**) nucleophilic attack of a water molecule on the carbonyl carbon atom of Asp333; (**b**) direct proton transfer from His523 to the negatively charged oxygen atom of epoxide; and (**c**) proton transfer from His523 to the negatively charged oxygen atom of epoxide through a water molecule and Tyr381; (**d**) AM1 PES corresponding to the nucleophilic attack of a water molecule and the proton transfer to His523 from hydroxyl intermediate. All distances are in Å and energies in kcal·mol^−1^.

The results obtained for mechanism B1 show that the nucleophilic attack of the water molecule on the carbonyl carbon atom of Asp333 takes place through a TS with an energy, related to the reactant complex, of 64.8 kcal·mol^−1^ at AM1 level and 57.3 kcal·mol^−1^ when corrected at M06-2X/6-31G+(d,p) level (see Figure 3a). In the case of the other two mechanisms, the proton transfer from His523 to the negatively charged oxygen atom of epoxide, either directly (mechanism B2) or through a water molecule and Tyr381 (mechanism B3), takes place with lower potential energy barriers: 4 kcal·mol^−1^ in the case of mechanism B2 (see Figure 3b), and through a stepwise mechanism of 2 and 4 kcal·mol^−1^ in the case of mechanism B3 (see Figure 3c). Afterwards, the nucleophilic attack of the water molecule and the proton transfer to His523 takes place. The corresponding PES, presented in Figure 3d, indicates that, as in mechanism B1, water attack occurs simultaneously with the proton transfer to Nε-His523 (Figure 3a) with a TS that would be 17.2 kcal·mol^−1^ over RC. Interestingly, it seems that the origin of the difference in energy barriers between mechanism B1 and B2 & B3 for this step lies not necessarily in the difference between the proton acceptors, but rather in the geometry of the transition states (TSs). Finally, the hydroxyl formation takes place in a single step in both mechanisms B1 or mechanism B2 & B3, with a potential energy barrier of 2.2 kcal·mol^−1^. All in all, the presented results allow us to conclude that hydroxyl formation from an oxyanion intermediate would preferentially occur through mechanism B2 or B3. The amount of tyrosinate formed during the nucleophilic attack was experimentally estimated to be 0.4 per enzyme molecule [41], indicating only partial ionization of the residues of the oxyanion hole and, implicitly, the relative relevance of mechanism B2 in the reaction mechanism.

### 2.2. t-DPPO Hydrolysis Catalyzed by CALB

As mentioned in the Introduction, the active site of CALB presents noticeable similarities with the active site of sEH. However, in the wild-type CALB the role of the nucleophile in its catalytic triad is played by a serine (Ser105) instead of an aspartate (Asp333) residue. In fact, as experimentally shown, the Asp333Ser variant of sEH loses its capability to catalyze the epoxide hydrolysis. Thus, it can be concluded that Ser105Asp CALB should perfectly mimic the active site of wild-type sEH. Apart from differences in the rest of the proteins’ scaffolds, the main difference between the active sites of sEH and Ser105Asp CALB is the residues involved in the formation of the oxyanion hole. Then, we can predict that the reaction in the latter should proceed through a path similar to mechanism B2 described for sEH. As we have already demonstrated, the role of the oxyanion hole in sEH is to stabilize the negatively charged oxygen of the intermediate, because the other possible role being a proton donor has already been excluded or does not present any energetic advantage (mechanism B3). Thus, the epoxide hydrolysis in the active site of wild-type CALB and Ser105Asp variant is studied assuming a reaction path similar to the most favorable one observed in sEH, mechanism B2.

**Figure 4:**
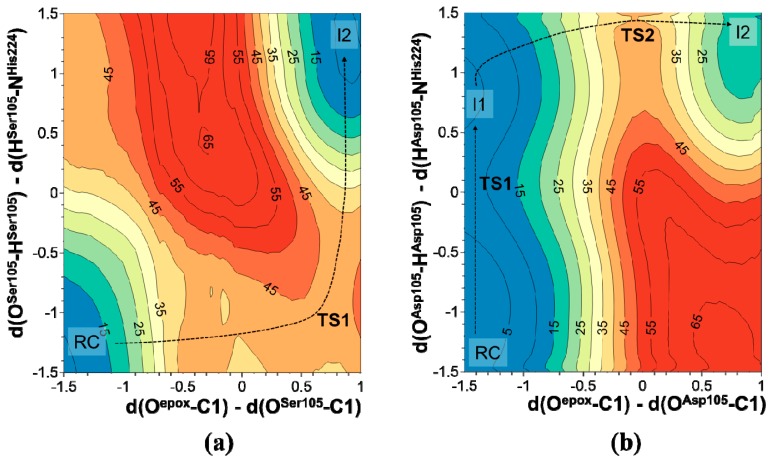
PES of the nucleophilic attack to C1 carbon atom of t-DPPO(1) by (**a**) Ser105 in wild-type CALB and (**b**) Asp105 in Ser105Asp CALB.

The resulting PESs of the nucleophilic attack to C1 carbon atom of t-DPPO(1) in wild-type and Ser105Asp CALB are shown in Figure 4. The corresponding PESs for the attack on the C2 carbon atom, as well as the PESs for the attack to C1 and C2 with the t-DPPO(2) orientation, are reported in Figures S1 and S2 of the Supplementary Materials, showing the same trend as that observed in Figure 4a,b. The analysis of PESs presented in Figure 4 shows a meaningful difference between the wild-type and Ser105Asp variants of CALB. First of all, in the case of wild-type CALB, the Ser105 attack to epoxide and the proton transfer to Nε-His523 take place in a concerted way while, in mutated CALB, attack of Asp105 occurs after proton transfer in a step-wise manner. Moreover, as presented in Table 2, the results show how, in agreement with the results obtained for the epoxide hydrolysis in the active site of sEH, the small differences in the energy barriers between different orientations of the substrate and the two possible nucleophilic attacks to C1 or C2 do not allow us to pinpoint any preference for the formation of one conformer. Consequently, it appears that quantum cluster models present limitations for subtle differences between the energy of conformers in this system. It is important to point out that, considering the good agreement between the trends obtained at the AM1 and M06-2X level of theory for the reaction studied in the sEH, and taking into account the high computational cost of repeating the calculations for this system at a higher level of theory, we report values obtained at the semi-empirical level. It is, then, reasonable to predict that barriers would decrease dramatically if computed at the DFT level of theory.

**Table 2:** Relative potential energies obtained at AM1 level related to reactant complex (RC) for the nucleophilic attack of Asp105 in Ser105Asp CALB on epoxide carbons C1 or C2 in two different orientations of substrate. Values are given in kcal·mol^−1^.

	(1)-DPPO		(2)-DPPO	
	C1	C2	C1	C2
**RC**	0.0	0.0	0.0	0.0
**TS1**	9.8	9.8	7.0	7.0
**I1**	2.6	2.6	−0.5	−0.5
**I1**	−0.8	0.3	2.0	−1.8
**TS2**	39.5	43.0	44.8	42.1

**Figure 5:**
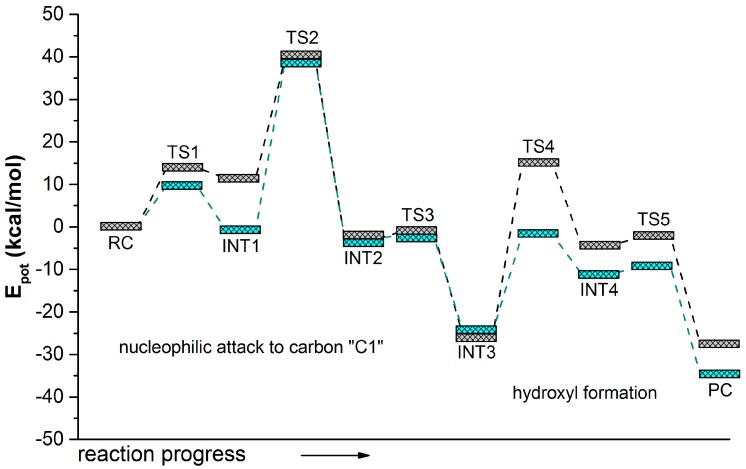
Potential energy profiles computed at the AM1 level for t-DPPO(1) hydrolysis catalyzed by sEH (**grey line**) and by Ser105Asp CALB (**green line**). The reaction corresponds to the catalytic attack on the C1 carbon of epoxide.

**Figure 6:**
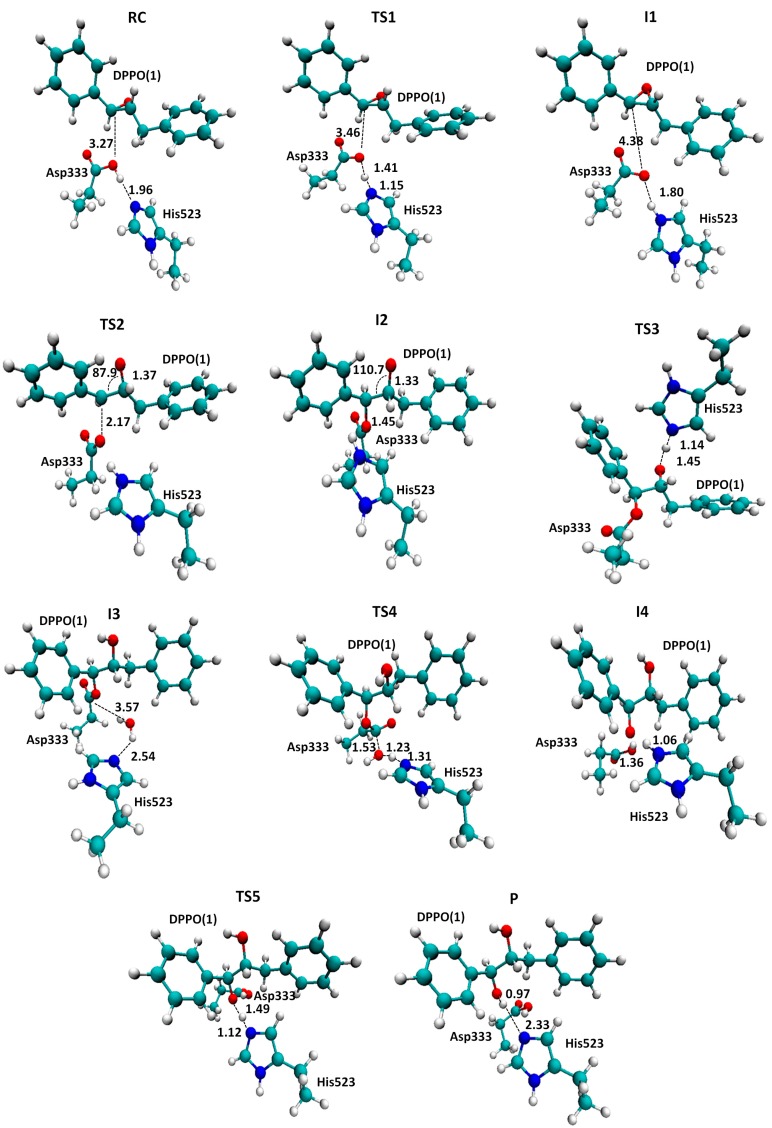
Localized structures for hydrolysis reaction of t-DPPO(1) catalyzed by sEH. Key inter-atomic distances are reported in Å. Tyr381 and Tyr465, forming the oxyanion hole, are not displayed for the purposes of clarity.

**Figure 7:**
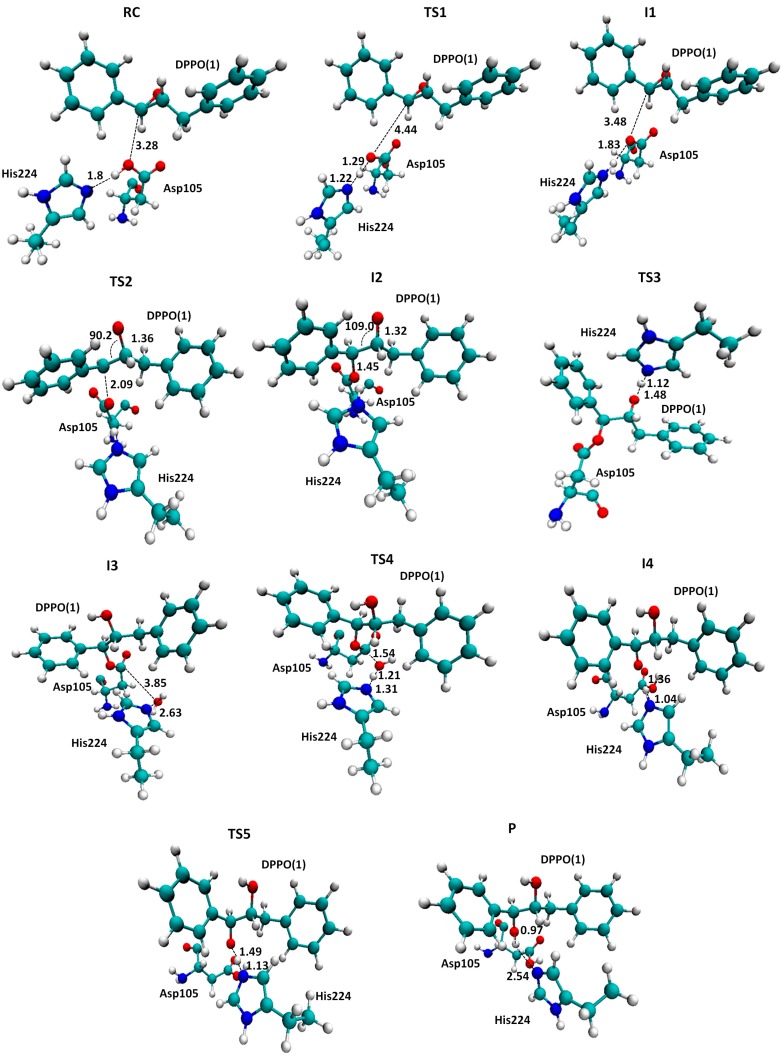
Localized structures for hydrolysis reaction of t-DPPO(1) catalyzed by Ser105Asp CALB. Key inter-atomic distances are reported in Å. Thr40 and Gln106, forming the oxyanion hole, are not displayed for the purposes of clarity.

In order to compare the pathway of the reaction catalyzed by she or by the Ser105Asp variant of CALB, the rest of the steps from INT3 to PC were explored following the same strategy as the one employed in the study of mechanism B2 & B3 of the reaction catalyzed by sEH. The full energy profiles are summarized in Figure 5, while a ball and stick representation of the stationary point structures is shown in Figure 6 and Figure 7, where key inter-atomic distances and angles are reported. The first conclusion that can be derived from an analysis of Figure 5 is that both reaction paths are quite similar, regarding the number of steps and the kind of transformations taking place in each step. Nevertheless, while the rate-limiting step for hydrolysis of t-DPPO(1) catalyzed by sEH corresponds to the fourth step, the proton transfer from the water molecule to His523 with concomitant attacking of the oxygen water molecule to Asp333 and breaking the bond established with the epoxide (TS4(B2 & B3) in Scheme 4), in the case of the reaction catalyzed by Ser105Asp CALB, the barrier of this step is dramatically reduced (see the corresponding PES in Figure S3 of the Supplementary Materials) and the rate-limiting step is then associated with the nucleophilic attack of the aspartate residue on the carbon atom of the epoxide ring, the breaking of the epoxide three-membered ring, and the stabilization of the negative charge developed in the oxygen atom by the oxyanion hole.

## 3. Computational Methods

The theoretical study of possible t-DPPO epoxide hydrolysis mechanisms has been performed within two different active sites models, as presented in Scheme 1. Models were prepared based on available Protein Data Bank crystal structures of sEH (PDB ID 1EK1) [28] and CALB (PDB ID 1TCA) [42] enzymes. Mutation of Ser105 to Asp in the CALB active site was achieved by using Discovery Studio 3.5 [43]. The cluster models, depicted in Scheme 1, have been selected to mimic the conserved catalytic triad and oxyanion hole found in the active site of these two α/β hydrolases, which have shown significant activity for epoxide hydrolysis reactions. Thus, in the model of the active site of sEH, the substrate is surrounded by Asp333, His523, Asp495, Tyr381, and Tyr465. All residues are saturated in Cα positions. The PESs for the different mechanism have been obtained at the AM1 semi-empirical level [44]. The highly parametrized M06-2X [45,46] hybrid functional was selected in order to improve the limitation of the AM1 method. The 6-31+G** basis set was used for the DFT calculations. After localizing the stationary points, frequency calculations were carried out to verify that the structures represent true minima or first-order saddle points on the gas phase PESs. Once first-order saddle points were located and characterized, the Intrinsic Reaction Coordinate (IRC) path was traced down from the saddle points to the corresponding minima using the full gradient vector. The global r.m.s. residual gradient in the optimized structures was always less than 0.04 kcal·mol^−1^·Å^−1^. It is important to note that no constraints were applied to any of the geometry optimizations. Although allowing more reliable energetics, this implies that possible artifacts, such as odd interaction complexes, can be obtained. Thus, proper orientation of the different structures in the starting point structures is a crucial step in the computational protocol. Also, keeping in mind that the reaction under study is a multi-step process, IRC calculations traced forward from a TS structure do not necessarily converge in the end of the backwards path traced from the following IRC. In this sense, efforts have been made to get a converged result; otherwise, the minimum energy structure, belonging to the reaction path, was selected. All AM1 calculations were performed with MOPAC2007 [47], while DFT calculations were with Gaussian 09 [48].

## 4. Conclusions

The primary reaction of the sEH, the hydrolysis of epoxides, has been studied by means of the AM1 semi-empirical method and the M06-2X hybrid functional, using a cluster model to mimic its active site. Using this reaction as a template, the secondary activity of wild-type CALB as an epoxidase has been studied and the comparison with the reaction on sEH has suggested a mutation of one residue of the active site that could improve the catalytic activity of CALB. Indeed, our results carried out with t-DPPO as a substrate suggest that the multi-step mechanism that Ser105Asp employs to hydrolyze t-DPPO is similar to the one used by sEH. Our predicted rate limiting step of sEH is in agreement with the pre-steady-state kinetic analysis of epoxide hydration catalyzed by mEH performed by Tseng *et al.* [49], who concluded that the rate of hydrolysis of the hydroxyl alkyl-enzyme intermediate was far slower than the rate of its formation. When the reaction is studied in the active site of the Ser105Asp CALB, our calculations show that the barrier of this step is dramatically reduced and the step associated to the nucleophilic attack of the aspartate residue to the carbon atom of the epoxide ring becomes the rate-limiting step. The barrier of this step is almost equivalent in both sEH and Ser105Asp CALB. Finally, any attempt to perform the nucleophilic attack by a possible water molecule in the active site of the sEH results in higher energy barriers, thus discarding this alternative mechanism. At this point, we must keep in mind that the values of the relative energies of the stationary points can be shifted when including the effect of the full protein. Nevertheless, since a comparative study has been carried out, in general the consequences should not be too dramatic.

From the computational point of view, our results, obtained at the AM1 level, follow the same trend as the ones recomputed at a higher level of theory, M06-2X with the 6-31+G** basis set. These latter values are, in fact, quite close to the barriers that can be deduced from experimentally measured rate constant in related enzymatic processes. Nevertheless, it appears that the stereo-selectivity of the enzyme, as a result of the nucleophilic attack taking place on one or the other carbon atom of the epoxide, cannot be predicted from simulations based on reduced models. Thus, further studies should be carried out in the future with more complex and realistic models such as the ones based on the use of hybrid QM/MM models. Information derived from the present study will be of great help in setting up the model, in determining the size and level of theory to be employed in the QM region, and in the analysis of the role of the key residues of the active site.

## Supplementary Materials

Supplementary materials can be accessed at: http://www.mdpi.com/1420-3049/20/10/17789/s1.
