# Supplementary material for: Is Promiscuous CALB a Good Scaffold for Designing New Epoxidases?
Source: Molecules. 2015 Sep 25;20(10):17789–806. doi: 10.3390/molecules201017789 (PMC6331936; doi:10.3390/molecules201017789)
Supplement: Supplementary file 1 [file molecules-20-17789-s001.pdf]

Supplementary Material

Table S1. pKa values of key residues in the active site of sEH and Ser105Asp CALB as computed with PROPKA program, compared with their standard values in solution.

	Residue	pKa in Enzyme	Standard pKa
**sEH**	Asp 333	10.31	3.80
	Asp 495	9.08	3.80
	His 523	1.23	6.50
	Tyr 465	18.12	10.00
	Tyr 381	14.82	10.00
**Ser105Asp CALB**	Asp 105	11.41	3.80
	His 224	5.19	6.50
	Asp 187	2.80	3.80

Table S2. Important distances for nucleophilic attack of water to C1 of DPPO(1) catalyzed by sEH. Values are given in Å.

Distances	RC	TS1	I1	TS2	PC
$H\delta^{His523}$–$N\delta^{His523}$	1.01	1.01	1.04(1.03)	1.02	1.01
$H\delta^{His523}$–O^{Asp495}	1.95	1.90	1.78(1.82)	1.85	1.94
O^{epox}–$C1^{epox}$	1.45	2.17	2.41(2.41)	2.41	2.42
O^{epox}–$C2^{epox}$	1.45	1.34	1.33(1.35)	1.37	1.43
O^{epox}–H^{Tyr381}	2.13	1.94	1.88(1.89)	1.92	2.10
O^{epox}–H^{Tyr465}	2.15	1.99	1.94(1.92)	1.97	2.23

Table S3. Important distances for nucleophilic attack of Aspartate to C1of DPPO(1) catalyzed by sEH and Ser105CALB. Values are given in Å.

Distances	RC		TS1		I1		TS2		I2	
	Wild Type sEH	Ser105Asp CALB	Wild Type sEH	Ser105Asp CALB	Wild Type sEH	Ser105Asp CALB	Wild Type sEH	Ser105Asp CALB	Wild Type sEH	Ser105Asp CALB
O^{Asp333}–H^{Asp333}	0.98	0.99	1.41	1.29	1.80(1.83)	1.83 (2.23)	1.88	1.90	2.45	2.30
H^{Asp333}–$N\varepsilon^{His523}$	1.96	1.87	1.15	1.22	1.03(1.03)	1.02 (1.02)	1.02	1.01	1.00	1.00
$H\delta^{His523}$–$N\delta^{His523}$	1.01	1.02	1.03	1.03	1.03(1.04)	1.04 (1.03)	1.04	1.04	1.04	1.04
$H\delta^{His523}$–O^{Asp495}	1.93	2.02	1.85	1.96	1.80(1.79)	1.98(1.95)	1.78	1.92	1.77	1.93
O^{Asp333}–$C1^{epox}$	3.27	3.28	3.46	4.44	4.38(3.26)	3.48 (3.94)	2.17	2.09	1.45	1.45
O^{Asp333}–$C2^{epox}$	3.58	4.31	3.73	5.60	4.10(3.56)	4.41 (4.06)	2.93	2.88	2.43	2.46
O^{epox}–$C1^{epox}$	1.45	1.44	1.45	1.45	1.45(1.45)	1.44 (1.44)	1.99	2.03	2.40	2.38
O^{epox}–$C2^{epox}$	1.44	1.44	1.44	1.44	1.45(1.44)	1.44 (1.44)	1.37	1.36	1.33	1.32
O^{epox}–H^{Tyr381}	2.13	2.25	2.12	2.24	2.14(2.12)	2.24(2.20)	1.98	1.99	1.90	1.91
O^{epox}–H^{Tyr465}	2.14	2.20	2.14	2.21	2.17(2.13)	2.19(2.36)	1.97	2.20	1.92	2.19

Table S4. Important distances for nucleophilic attack of Aspartate to C2 of DPPO(1) catalyzed by sEH and Ser105CALB.Values are given in Å.

Distances	RC		TS1		I1		TS2		I2	
	Wild Type sEH	Ser105Asp CALB	Wild Type sEH	Ser105Asp CALB	Wild Type sEH	Ser105Asp CALB	Wild Type sEH	Ser105Asp CALB	Wild Type sEH	Ser105Asp CALB
O^{Asp333}–H^{Asp333}	0.98	0.99	1.41	1.29	1.80(2.05)	1.83 (1.86)	2.10	1.95	2.44	2.30
H^{Asp333}–$N\varepsilon^{His523}$	1.96	1.87	1.15	1.22	1.03(1.01)	1.02 (1.02)	1.01	1.01	1.00	1.00
$H\delta^{His523}$–$N\delta^{His523}$	1.01	1.02	1.03	1.03	1.03(1.03)	1.04 (1.04)	1.04	1.04	1.04	1.04
$H\delta^{His523}$–O^{Asp495}	1.93	2.02	1.85	1.96	1.80()	1.98(1.92)	1.80	1.93	1.77	1.95
O^{Asp333}–$C1^{epox}$	3.27	3.28	3.46	4.44	4.38(3.98)	3.48 (4.82)	3.02	2.93	2.54	2.55
O^{Asp333}–$C2^{epox}$	3.58	4.31	3.76	5.60	4.10(3.15)	4.41 (4.07)	2.11	1.98	1.44	1.45
O^{epox}–$C1^{epox}$	1.45	1.44	1.45	1.45	1.45(1.45)	1.44 (1.44)	1.38	1.35	1.33	1.32
O^{epox}–$C2^{epox}$	1.44	1.44	1.44	1.44	1.45(1.45)	1.44 (1.44)	1.96	2.05	2.37	2.42
O^{epox}–H^{Tyr381}	2.13	2.25	2.12	2.24	2.14(2.15)	2.24(4.50)	1.98	4.17	1.90	1.93
O^{epox}–H^{Tyr465}	2.14	2.20	2.14	2.21	2.17(2.13)	2.19(6.35)	1.99	5.69	1.86	2.26

Table S5. Important distances for nucleophilic attack of Aspartate to C1 of DPPO(2) catalyzed by sEH and Ser105CALB.Values are given in Å.

Distances	RC		TS1		I1		TS2		I2	
	Wild Type sEH	Ser105Asp CALB	Wild Type sEH	Ser105Asp CALB	Wild Type sEH	Ser105Asp CALB	Wild Type sEH	Ser105Asp CALB	Wild Type sEH	Ser105Asp CALB
O^{Asp333}–H^{Asp333}	0.98	0.99	1.43	1.26	1.79(1.84)	1.86(1.87)	1.90	1.93	2.07	2.12
H^{Asp333}–$N\varepsilon^{His523}$	2.12	1.87	1.14	1.25	1.03(1.02)	1.02(1.02)	1.02	1.01	1.00	1.00
$H\delta^{His523}$–$N\delta^{His523}$	1.01	1.02	1.03	1.02	1.04(1.04)	1.04(1.04)	1.04	1.04	1.05	1.04
$H\delta^{His523}$–O^{Asp495}	1.91	2.01	1.85	1.95	1.80(1.79)	1.90(2.01)	1.77	1.91	1.74	1.90
O^{Asp333}–$C1^{epox}$	3.63	4.56	3.96	4.83	3.47(3.70)	4.62(3.78)	2.09	1.99	1.45	1.46
O^{Asp333}–$C2^{epox}$	3.77	3.48	3.81	3.76	3.65(3.25)	3.62(3.85)	2.97	2.95	2.58	2.50
O^{epox}–$C1^{epox}$	1.44	1.44	1.45	1.44	1.44(1.45)	1.44(1.44)	1.96	2.08	2.37	2.43
O^{epox}–$C2^{epox}$	1.45	1.44	1.45	1.44	1.45(1.45)	1.44(1.44)	1.38	1.34	1.33	1.32
O^{epox}–H^{Tyr381}	2.15	2.46	2.15	2.50	2.13(2.15)	2.65(5.60)	2.00	3.99	1.93	1.91
O^{epox}–H^{Tyr465}	2.13	3.69	2.12	3.56	2.12(2.13)	3.81(6.43)	1.98	5.57	1.89	2.18

Table S6. Important distances for nucleophilic attack of Aspartate to C2 of DPPO(2) catalyzed by sEH and Ser105CALB.Values are given in Å.

Distances	RC		TS1		I1		TS2		I2	
	Wild Type sEH	Ser105Asp CALB	Wild Type sEH	Ser105Asp CALB	Wild Type sEH	Ser105Asp CALB	Wild Type sEH	Ser105Asp CALB	Wild Type sEH	Ser105Asp CALB
O^{Asp333}–H^{Asp333}	0.98	0.99	1.43	1.26	1.79(1.77)	1.86(2.27)	1.88	2.04	2.16	2.21
H^{Asp333}–$N\varepsilon^{His523}$	2.12	1.87	1.14	1.25	1.03(1.03)	1.02(1.02)	1.02	1.01	1.01	1.00
$H\delta^{His523}$–$N\delta^{His523}$	1.01	1.02	1.03	1.02	1.04(1.03)	1.04(1.03)	1.04	1.04	1.04	1.04
$H\delta^{His523}$–O^{Asp495}	1.91	2.01	1.85	1.95	1.80(1.80)	1.90(1.78)	1.78	1.94	1.76	1.76
O^{Asp333}–$C1^{epox}$	3.63	4.56	3.96	4.83	3.47(3.61)	4.62(4.45)	2.94	2.90	2.43	2.51
O^{Asp333}–$C2^{epox}$	3.77	3.48	3.81	3.76	3.65(2.92)	3.62(3.46)	2.11	2.00	1.45	1.44
O^{epox}–$C1^{epox}$	1.44	1.44	1.45	1.44	1.44(1.45)	1.44(1.44)	1.38	1.36	1.33	1.32
O^{epox}–$C2^{epox}$	1.45	1.44	1.45	1.44	1.45(1.45)	1.44(1.45)	1.96	2.01	2.39	2.36
O^{epox}–H^{Tyr381}	2.15	2.46	2.15	2.50	2.13(2.15)	2.65(2.14)	2.00	2.09	1.94	1.94
O^{epox}–H^{Tyr465}	2.13	3.69	2.12	3.56	2.12(2.15)	3.81(2.26)	1.98	2.15	1.89	2.13

Table S7. Important distances for hydroxyl formation for DPPO(1) catalyzed by sEH. Values are given in Å.

Distances	I2		TS3(B1)		I3		TS4(B1)		P	
	Wild Type sEH	Ser105Asp CALB	Wild Type sEH	Ser105Asp CALB	Wild Type sEH	Ser105Asp CALB	Wild Type sEH	Ser105Asp CALB	Wild Type sEH	Ser105Asp CALB
O^{Asp333}–H^{Asp333}	2.22	2.27	2.18	2.66	2.09(4.86)	4.66 (4.66)	4.64	4.56	3.60	4.08
H^{Asp333}–$N\varepsilon^{His523}$	1.00	1.00	1.00	1.00	1.00(1.04)	1.04 (1.05)	1.11	1.10	2.59	2.60
$H\delta^{His523}$–$N\delta^{His523}$	1.04	1.04	1.04	1.05	1.04(1.03)	1.04 (1.03)	1.03	1.03	1.01	1.01
$H\delta^{His523}$–O^{Asp495}	1.76	1.76	1.75	1.73	1.76(1.81)	1.79(1.94)	1.83	1.96	1.98	2.04
O^{Asp333}–$C1^{epox}$	1.44	1.45	1.43	1.45	1.43(1.43)	1.43 (1.42)	1.43	1.43	1.43	1.42
O^{Asp333}–$C2^{epox}$	2.48	2.48	2.50	2.44	2.52(2.52)	2.84 (2.49)	2.48	2.48	2.45	2.44
O^{epox}–$C1^{epox}$	2.37	2.37	2.38	2.40	2.38(2.38)	2.43 (2.40)	2.38	2.40	2.41	2.41
O^{epox}–$C2^{epox}$	1.33	1.32	1.33	1.32	1.33(1.36)	1.34 (1.34)	1.37	1.36	1.43	1.42
O^{epox}–H^{Tyr381}	1.88	1.92	1.90	1.91	1.90(1.88)	1.96(1.93)	1.92	1.96	2.06	2.10
O^{epox}–H^{Tyr465}	1.91	2.16	1.92	2.20	1.90(1.97)	2.17(2.14)	2.02	2.15	2.29	2.29
O^{wat}–C^{Asp333}	3.08	3.56	1.54	1.53	1.36(1.36)	1.36 (1.36)	1.36	1.36	1.36	1.36
H^{wat}–O^{Asp333}	3.31	4.26	1.30	1.30	0.97(0.97)	0.97 (0.97)	0.97	0.97	0.97	0.97

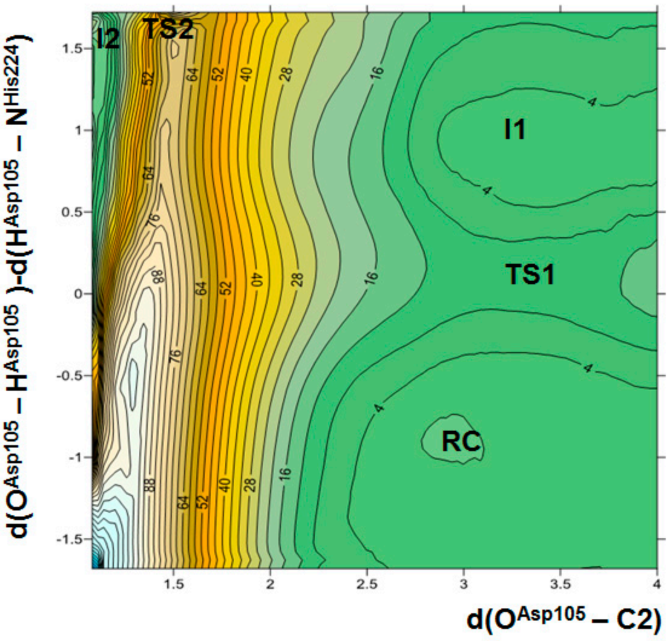

Figure S1. PES of the nucleophilic attack to C2 carbon atom of t-DPPO(1) in Ser105Asp CALB. All distances are in Å and energies in kcal·mol^{-1}.

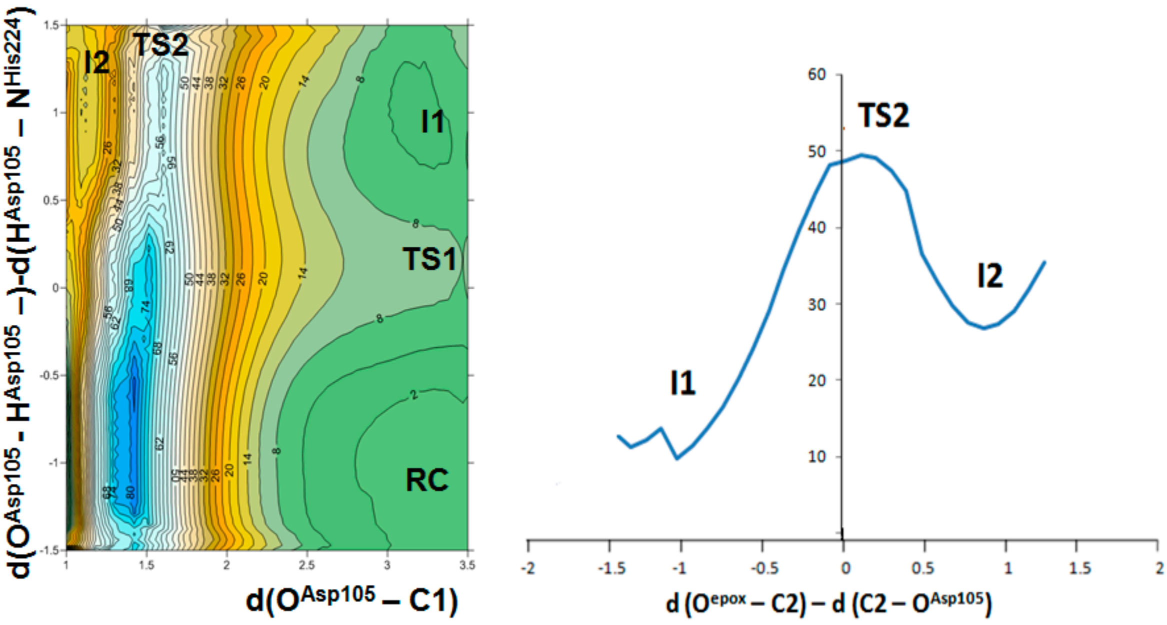

(**a**) (**b**)

Figure S2. PES of the nucleophilic attack to C1 carbon atom of t-DPPO(2) in Ser105Asp CALB (**a**) and PES of the nucleophilic attack to C2 carbon atom (**b**). All distances are in Å and energies in kcal·mol^{-1}.

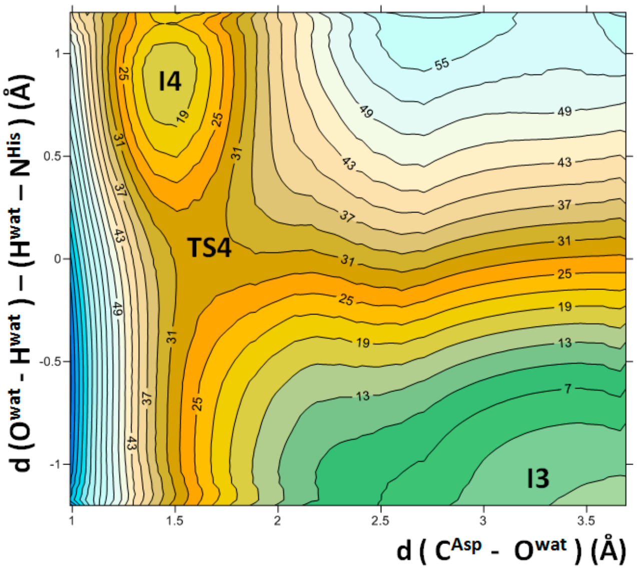

Figure S3. PES of the I3 to I4 step of t-DPPO(1) hydrolysis in Ser105Asp CALB. Distances are in Å and energies in kcal·mol^{-1}.
